# On the Use of Iodine in Opacity of the Cornea

**Published:** 1841-09-11

**Authors:** William S. Helmuth


*On the use of Iodine in Opacity of the Cornea.*
By William S. Helmuth, M.D.

In the thirty-first number of the Examiner of the present year, there is among the foreign items, a notice of a case of corneal opacity, successfully treated by iodine by Dr. Lohsse. The successful use of the same remedy in four cases of corneal opacity, within the past twelve months, enables me to offer further evidence of the efficacy of the remedy.

In the beginning of September, 1840, when requested to visit Miss E. V., I found the left eye presenting the usual appearances of a severe ophthalmia, together with corneal opacity; in the course of a few weeks the right eye was attacked in a similar manner. The acute inflammatory symptoms were subdued by leeching, saline purgatives, antimonials, and other antiphlogistic measures. The opacity alone remaining, iodine was prescribed as follows:

℞. Iodine, ℈j.

Hydriodat. Potassæ, ℈ij.

Aquæ fluvialis, ℥j.

Fiat. solut.

Dose. Five drops ter die.

This dose was gradually increased during the treatment, till fourteen drops were taken each time.

There was also directed at the same time,

℞. Belladonnæ, gr. i.
Aquæ distil. ℥i.
M. ft. collyr. To be applied several times during the day to the eyeball.

The prognosis was for awhile very unfavorable, there being at one time a deposit of pus in the cornea, from which radiated red vessels, and the general appearance of the eye was that of structural derangement. Dr. Thos. T. Hewson was in consultation, and continued his visits till the alarming symptoms disappeared, and the opacity was decidedly diminishing. It is not even yet entirely gone, though it so steadily lessens that a complete cure is confidently expected; which anticipation is rendered the more certain from a comparison with the progress and fortunate termination of the cases which will be next noticed. The only means at present used is the following ointment:

℞. Iodine, gr. iss.

Axung. Porcinæ, ʒss.

M. fiat. unguent.

A small portion to be introduced under the eyelids once or twice a day.

The next case was that of Miss H. N. ætat. 23 years, whom I first visited in February, 1841. The inflammation also in this case commenced in one eye, and after some weeks attacked the other. Though less inflammatory than the preceding case, its progress through the different stages was similar, and in five months yielded to the same treatment. After the cornea regained its transparency, objects occasionally appeared indistinct, for which the 1-16th part of a grain of strichnine was taken at bed time, the repetition of which for a few nights rendered vision much clearer.

The next case was that of Miss F. aged 16 years. The disease was confined to one eye, and although the opacity was quite as much as in the last mentioned case, the accompanying conjunctival inflammation was milder. The iodine was used as in the other cases, and the cure was completely established in a little more than three months; my first visit being on April the 7th, and on the 25th of July last the opacity was entirely away.

The fourth case was that of a boy aged about four years. But one of the eyes was effected. At the commencement there was acute general inflammation of the part, which rendered leeching, &c., necessary, after which the iodine, in doses, proportioned to his age, was directed. The disease commenced in the middle of June, and on the 3d of August last the opacity no longer existed.

During the treatment of each of the preceding cases, when the opacity alone remained, there was substituted for a strict regimen, a more generous diet, and the eyes were protected by a green shade only, and even this defence was often neglected: nor did the improvement appear to be retarded, nor the disease increased by the exposure.

I am not informed whether there existed any unusual disposition to this form of disease during the last year. Nor could I discover any sufficient cause for the disease in the individuals whose cases have just been stated. The patients were at the time of the attack, and had been previously, in perfect health; neither had they, as far as I could ascertain, any constitutional or hereditary taint. Where both eyes were affected, the disease first appeared in the left, and when one eye only was effected it was the left.

The opacity was of that variety denominated by Beer Macula corneæ simplex, obscuratio. It did not appear after the conjunctival inflammation, but simultaneously with it.

In conclusion I may remark, in relation to the employment of iodine, that, after a good deal of experience in its employment in a variety of diseases, both in large and frequent doses, and for a long time continued, especially in scrofula, I have not witnessed those ill consequences which it is said it sometimes occasions.

